# Identification and Expression of Immunogenic Mimotopes of *C. hepaticus* Using an *E. coli*-Based Surface Display System

**DOI:** 10.3390/vaccines14040298

**Published:** 2026-03-26

**Authors:** Chaitanya Gottapu, Lekshmi K. Edison, Roshen N. Neelawala, Varsha Bommineni, Gary D. Butcher, Bikash Sahay, Subhashinie Kariyawasam

**Affiliations:** 1 Comparative, Diagnostic, and Population Medicine, College of Veterinary Medicine, University of Florida, Gainesville, FL 32610, USA; cgottapu@ufl.edu (C.G.); edison.le@ufl.edu (L.K.E.); roshen.neelawala@ufl.edu (R.N.N.); vbommineni@ufl.edu (V.B.); 2 Department of Large Animal Clinical Sciences, College of Veterinary Medicine, University of Florida, Gainesville, FL 32610, USA; butcher@ufl.edu; 3 Department of Infectious Diseases and Immunology, College of Veterinary Medicine, University of Florida, Gainesville, FL 32610, USA; sahayb@ufl.edu

**Keywords:** *Campylobacter hepaticus*, flagella, major outer membrane protein, mimotopes, outer membrane protein C, surface display system, vaccines

## Abstract

**Background/Objectives**: Spotty liver disease (SLD), caused by *Campylobacter hepaticus*, is an emerging disease that leads to substantial production losses in the egg industry. The shift toward antibiotic-free and cage-free production systems has further intensified the impact of SLD. The current control measures largely rely on autogenous killed vaccines; however, their use is constrained by the slow and fastidious growth of *C. hepaticus* and inconsistent efficacy. To overcome these limitations, this study aimed to identify immunogenic mimotopes as vaccine candidates and express them on the surface of an avian pathogenic *Escherichia coli* (APEC) vector. **Methods**: To identify immunogenic mimotopes, Ph.D.-12 phage display peptide library was screened using the hyperimmune serum raised against killed whole-cell *C. hepaticus* in specific pathogen-free chickens. Subsequently, the outer membrane protein C (OmpC) of *E. coli* was used as a scaffold for constructing a surface display library. A single restriction site, *Pst*I, located in the seventh external loop of OmpC, was strategically utilized to insert each 12-amino-acid mimotope with a six-histidine (6xHis) tag sequence at its N-terminus, generating *ompC* + mimotope fusion constructs. These constructs were cloned into the inducible expression vector pTrc and electroporated into an *E. coli* DH5α ∆*ompC* strain, which lacked *ompC*. The surface expression of the mimotopes was confirmed in vitro. The verified *ompC* + mimotope constructs were subsequently subcloned into the pYA3422 constitutive expression vector and electroporated into the APEC PSUO78 ∆*aroA* ∆*asd* vaccine vector strain. A chicken vaccination–challenge trial was conducted using nine groups of chickens, including an unvaccinated challenged control and an unvaccinated–unchallenged negative control. Each experimental group received a mixture of two recombinant *E. coli* strains carrying different mimotopes at a dose of 1 × 10^9^ CFU, which were administered orally twice at 16 and 18 weeks of age. **Results**: Fourteen immunogenic mimotopes corresponding to 13 different *C. hepaticus* proteins were identified as potential vaccine candidates. The expression of these mimotopes on the surface of the *E. coli* was successfully demonstrated using the OmpC-mediated surface display system. Of the 14 mimotopes tested, two flagellar-related peptides and one major outer membrane protein (MOMP)-derived peptide elicited significant immune responses and conferred protection against the *C. hepaticus* challenge. **Conclusions**: We successfully developed a functional *E. coli* surface display system that was capable of expressing 12-amino-acid mimotopes of *C. hepaticus*, providing a robust platform for evaluating vaccine candidates against SLD. Immunogenicity and efficacy studies in chickens demonstrated that three identified mimotopes conferred protection against *C. hepaticus* colonization of the bile and liver. Future in vivo investigations are necessary to develop and evaluate the immunogenicity and protective efficacy of a multivalent mimotope vaccine consisting of three identified mimotopes against both *C. hepaticus* and APEC, utilizing the Δ*aroA* Δ*asd* APEC PSU078 strain as the vaccine vector.

## 1. Introduction

The United States of America is the top global poultry meat producer and the second-largest egg producer by volume [1]. The layer industry is continuously advancing to enhance both production efficiency and quality parameters [2]. The consumer perception of quality is shifting towards production and processing attributes, which is reflected by forecasted annual growth of more than 6% in sustainable foods from 2021 to 2028 [3,4]. Although many consumers still avoid organic eggs due to their higher cost compared to those from conventionally caged hens, there is growing acceptance and preference for cage-free eggs, which better align with ethical and welfare considerations [5,6]. Reflecting this shift, major egg retailers in the United States have committed to transitioning their supply chains to 100% cage-free eggs in the coming years [7]. This shift toward cage-free (barn and aviary) and free-range systems, driven by consumer and regulatory demands, creates a new set of health management challenges for egg producers, such as an increased incidence of diseases and a greater exposure to pathogens, particularly those spread via the fecal–oral route [8,9]. Among these emerging health threats, spotty liver disease (SLD) has become a major concern, with recent increases in outbreaks directly associated with the transition from conventional cage systems to cage-free production [10].

The recently emerged *Campylobacter* species, *Campylobacter hepaticus*, causes spotty liver disease (SLD) in layer hens, predominantly during peak egg production [11,12]. *C. hepaticus*, a novel, fastidious bacterium, was first isolated from affected flocks in the United Kingdom by Crawshaw et al. in 2015 [13]. This organism has been formally isolated, characterized, and designated as a new species, *C. hepaticus*, by Van et al. in 2016 [14]. In recent years, SLD outbreaks have been increasingly reported across various geographic regions of the United States, and confirmed cases have been documented in multiple states, including Iowa, Florida, and Georgia [15,16,17,18].

This disease is characterized by multifocal hepatic lesions (spots on the liver surface) with high mortality rates (up to 15%) and production losses (up to 35%) [19]. SLD predominantly affects free-range and floor-raised chicken flocks, although sporadic cases have been reported in caged systems [20,21]. Currently, there are no approved therapeutic options for SLD, and tetracyclines are typically administered during SLD outbreaks [22]. Feed additives such as biochar and isoquinoline alkaloids (IQA), along with acidifying products such as apple cider vinegar or citric acid, have shown partial success in reducing *C. hepaticus* loads under experimental challenges [22,23,24]. However, like other *Campylobacter* species, *C. hepaticus* exhibits antimicrobial resistance (AMR), which may lead to treatment failures [25,26]. In addition, moving towards antibiotic-free commercial egg production requires a logical solution to control SLD [12,27].

Recognizing the urgent need for sustainable, non-antibiotic control strategies, vaccine development has become a major focus in the poultry industry [28]. Although traditional inactivated whole-cell vaccines can elicit strong antibody responses, the fastidious nature of *C. hepaticus* poses significant technical challenges in large-scale culture and vaccine production [12]. As an innovative solution for more targeted vaccine antigen delivery, advanced platforms such as phage display peptide libraries are being employed [29,30]. This technology allows for the high-throughput screening of hyperimmune sera raised against whole microorganisms to identify short peptide mimotopes that mimic the key protective epitopes of pathogens [30,31]. These mimotopes serve as molecular surrogates for native antigenic determinants, facilitating the precise identification of the most immunogenic targets for vaccine design [29].

To effectively present these mimotopes to the host immune system, surface display systems have been developed [32,33]. These systems enable the exposure of heterologous peptides or proteins on the bacterial surface, typically achieved through C- or N-terminal fusions [34,35]. Outer membrane proteins (OMPs) are present on bacterial surfaces and mediate direct interactions with the extracellular environment [36]. However, many native OMPs lack suitable anchoring motifs, and modifications at their termini often interfere with correct protein folding, membrane insertion, or stability of the carrier protein [37]. As OMPs generally require the entire structure for correct assembly, conventional fusion approaches can compromise their functionality. Sandwich fusion overcomes these limitations by inserting small epitopes into the external loops of the carrier protein, thereby preserving the structural integrity of the OMP while enabling a stable surface display of the inserted mimotopes [36]. This approach has been successfully applied to several bacterial display systems and has shown promise in vaccine development against diverse pathogens [38]. Among the different OMPs that were evaluated for surface display, OmpC is a particularly suitable candidate because it is a highly abundant porin with a well-characterized β-barrel structure that lends itself to genetic modifications [39]. Structurally, OmpC consists of 16 β-strands that form a barrel with eight extracellular loops, several of which have been shown to tolerate small insertions or deletions without disrupting the membrane insertion, folding, or stability [40]. Compared to other commonly studied OMPs, such as OmpA and OmpF, OmpC offers several advantages in displaying short 12-amino-acid mimotopes. Although OmpA is highly stable, it has limited surface-exposed loops, which limit the number of permissive sites for peptide insertion [33,36]. OmpF, which is structurally similar to OmpC, is less abundantly expressed under standard growth conditions, and its extracellular loops exhibit greater structural variability, potentially reducing the consistency of the surface display [41,42]. In contrast, OmpC combines high natural expression, structural stability, and well-defined permissive extracellular loops, providing an optimal platform for small peptide display while maintaining proper membrane localization and immunogenic exposure [40]. This study aimed to develop and confirm the surface expression of *C. hepaticus* mimotopes using an OmpC-based display system in the avian pathogenic *E. coli* (APEC) PSUO78 ∆*aroA* ∆*asd* double mutant strain and to evaluate the immunogenicity and protective efficacy of the vaccine constructs against SLD in a controlled chicken challenge model.

## 2. Materials and Methods

### 2.1. Bacterial Strains, Plasmids, and Culture Conditions

The bacterial strains, plasmids, and their respective growth conditions used in this study are listed in Table 1. The *C. hepaticus* strain FL2019SK1 is primarily used for antibody production. The *E. coli* K-12 strain (*fhuA2*Δ(*argF-lacZ*)U169 *phoA glnV44* Φ*80*Δ(*lacZ*)M15 *gyrA96 recA1 relA1 endA1 thi-1 hsdR17*) served as the parental strain for creating the OmpC mutant and establishing the surface display system. The APEC PSUO78 ∆*aroA*∆*asd* double mutant strain was used as a live vector for the delivery of *C. hepaticus* mimotopes as vaccine candidates. The lyophilized pTrc99a vector was purchased from Creative Biogene (Creative Biogene, Shirley, New York, NY, USA). The *ompC* gene fragment was introduced into the multiple cloning site (MCS) of pTrc99a vector using *Sal*I and *Hind*III restriction sites, and the resulting construct was electroporated into the *E. coli* DH5α ∆*ompC* strain. Expression of OmpC from the pTrc99a vector was induced at an optical density at 600 nm of 0.6 by adding isopropyl-D-1-thiogalactopyranoside (IPTG) (Thermo Fisher Scientific, Waltham, MA, USA) to a final concentration of 0.5 μM. The *C. hepaticus* strains USA1, USA5 and USA52 were used for challenging the vaccinated and unvaccinated control groups, which were cultured on Brucella agar plates supplemented with 5% laked horse blood agar (HBA) and incubated at 37 °C for 7 days under microaerophilic conditions using a Mitsubishi AnaeroPack-MicroAero gas generator (Thermo Fisher Scientific) for five days.

### 2.2. Preparation of Heat-Killed C. hepaticus

The *C. hepaticus* strain FL2019SK1 was streaked on Brucella agar plates supplemented with 5% laked horse blood agar (HBA) and incubated at 37 °C for 7 days under microaerophilic conditions using a Mitsubishi AnaeroPack-MicroAero gas generator (Thermo Fisher Scientific) for five days. The *C. hepaticus* colonies were confirmed via a polymerase chain reaction (PCR). The bacteria were harvested in a biosafety cabinet using phosphate-buffered saline (PBS, pH 7.2–7.4). The bacterial suspension was centrifuged at 8000× *g* for 10 min at 4 °C. The supernatant was discarded, and the pellet was washed twice by resuspending it in fresh sterile PBS, followed by centrifugation. For inactivation, the bacterial aliquots were heated at 60 °C for 60 min. To confirm that no viable bacteria were present, 100 µL aliquots were streaked onto the HBA plates and incubated microaerophilically at 37 °C for seven days. The final inoculum was prepared by combining the 10^9^ CFU equivalent of inactivated bacteria with Freund’s incomplete adjuvant (Sigma-Aldrich, St. Louis, MO, USA) in a 1:1 (*v*/*v*) ratio. A uniform emulsion was created by vigorously transferring the mixture between syringes 20–50 times.

### 2.3. Production of Hyperimmune Sera

For the antibody production, specific pathogen-free (SPF) female Leghorn chickens from Charles River Laboratories (Charles River Laboratories, Wilmington, MA, USA) were used. The commercial Hy-Line layer chickens that were confirmed to be free of SLD (via cloacal swab testing for *C. hepaticus*) and with no prior SLD history were included in the vaccine immunogenicity and protection trials. The chickens received either an antibiotic-free corn-based starter diet or a wheat/barley-based grower’s diet (Purina Mills, St. Louis, MO, USA) ad libitum. All of the chicken-related experiments adhered to the protocols approved by the University of Florida’s Institutional Animal Care and Use Committee (IACUC). To generate antibodies, five SPF chickens were intramuscularly inoculated with the *C. hepaticus* vaccine–adjuvant mixture at 12 and 14 weeks of age. Two weeks after the booster vaccination, blood was drawn from the wing vein. The serum was then separated and stored at −20 °C. The polyclonal antibodies were purified using the caprylic acid method [46].

### 2.4. Phage Display and Mimotope Identification

To identify the peptide mimotopes of *C. hepaticus* FL2019SK1, a Ph.D.-12 phage display peptide library (New England BioLabs, Ipswich, MA, USA), which contained linear 12-mer peptides fused to the M13 phage pIII coat protein, was screened using polyclonal antibodies raised against *C. hepaticus* FL2019SK1, according to the manufacturer’s instructions. Three rounds of biopanning were performed, and a high specificity was ensured by increasing the concentration of Tween-20 (Sigma-Aldrich, St. Louis, MO, USA) in the washing buffers and the number of washing steps in each successive round. The phage titers were measured using a plaque assay, and the output was cultured on Luria–Bertani (LB)/IPTG/5-bromo-4-chloro-3-indolyl β-D-galactopyranoside (X-gal) agar and incubated overnight at 37 °C. One hundred individual plaques were randomly selected from the plates, inoculated into a log-phase ER2738 solution (37 °C, 4.5 h), and centrifuged to collect the supernatant containing the phages for further analysis. The DNA templates of the positive clones were sequenced at Azenta Life Sciences (Azenta Life Sciences, South Plainfield, NJ, USA) using the provided primers. The resulting DNA sequences were translated into peptide sequences using the ExPASy Translate tool (https://web.expasy.org/translate/ on 3 July 2023) and compared with those of the sequenced *C. hepaticus* FL2019SK1 strain. Furthermore, the peptides were searched via BLASTP and PHI-BLAST against the National Center for Biotechnology Information (NCBI BLAST+ suite, version 2.16.0) *C. hepaticus*-encoded open reading frame (ORF) database to identify highly similar proteins, which were then subjected to subcellular localization prediction for further selection. To select mimotopes that would confer protection against a broad range of *C. hepaticus* strains, the DNA sequences of the selected epitopes were compared with those of sequenced *C. hepaticus* strains from the GenBank database. The subcellular localization of the proteins corresponding to the selected mimotopes was predicted using the Cell-PLoc (http://www.csbio.sjtu.edu.cn/bioinf/Cell-PLoc-2/ on 4 July 2023) and PSORTb (https://psort.org/psortb/ on 4 July 2023) databases. The proteins favored as vaccine candidates were those predicted to be surface-exposed or secreted, while those with more than one transmembrane helix (TMH) were discarded due to their unlikelihood of being transported beyond the bacterial inner membrane. Finally, the mimotopes that were identified as epitope regions of the *C. hepaticus* cell-surface-localized proteins that exhibited either high (≥five continuous amino acid residues) or moderate (≥three but <five continuous amino acid residues) sequence similarity were included in this study.

### 2.5. Construction of the E. coli DH5α ompC Mutant Strain

The lambda (λ) Red recombinase-based one-step gene inactivation protocol was used to create the *E. coli* DH5α *ompC* mutant strain [47]. Briefly, the Red helper plasmid pKD46 (ampicillin resistant), which encodes the λ Red recombinase, was introduced into the DH5α strain by electroporation. The positive colonies were confirmed by plasmid verification and stored at −80 °C as glycerol stocks. For recombination, a PCR-generated disruption cassette containing the chloramphenicol resistance (CmR) marker flanked by flippase recognition target (FRT) sites and homologous arms (~40–50 bp) corresponding to the target gene was electroporated into *E. coli* DH5α/pKD46 cells expressing λ Red recombinase. The recombinants were selected on antibiotic-containing media, and successful integration was confirmed using PCR. Then, pKD46 was cured by incubating the confirmed clones at 42 °C, followed by plating on a non-selective medium to verify plasmid loss, which is indicated by the absence of growth on the carbenicillin. For Cm cassette excision, the resultant mutant was transformed with the temperature-sensitive plasmid pCP20 expressing the FLP (flippase) recombinase, selected on carbenicillin plates, and incubated at 30 °C. The FLP recombinase-mediated recombination removed the FRT-flanked CmR cassette, as confirmed by a PCR, after which pCP20 was cured by incubating the transformants at 42 °C and passaging on the non-selective LB medium. The resultant unmarked *E. coli* DH5α Δ*ompC* strain was confirmed using a PCR.

### 2.6. Construction of the ompC-Based Surface Display System Using the E. coli DH5α ΔompC Strain to Display C. hepaticus Mimotopes on the Bacterial Surface

**Figure 1:**
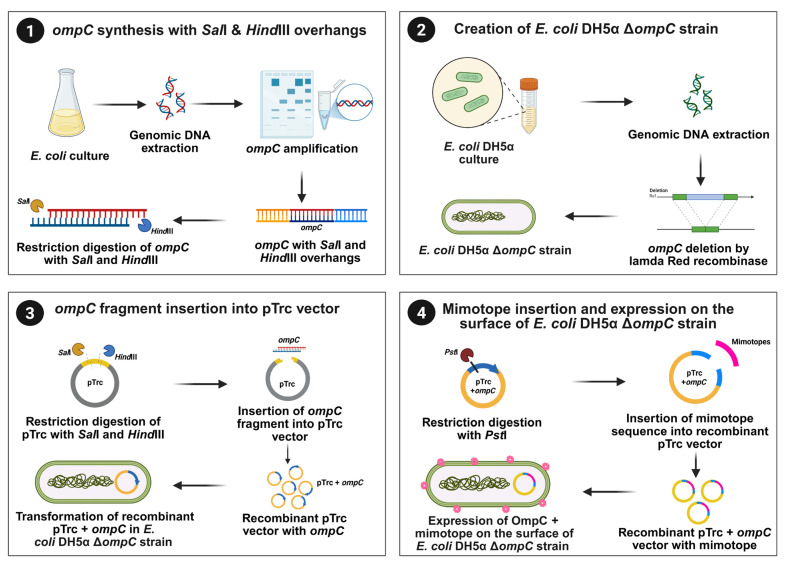
Stepwise construction of recombinant *E. coli* DH5α Δ*ompC* strain expressing an OmpC–mimotope fusion on the surface. (**1**) *ompC* synthesis with *Sal*I and *Hind*III overhangs: the genomic DNA was extracted from *E. coli*, and the *ompC* gene was amplified by PCR. The amplicon was digested with *Sal*I and *Hind*III to generate compatible cohesive ends for cloning. (**2**) Creation of the *E. coli* DH5α Δ*ompC* strain: the *ompC* gene was deleted from *E. coli* DH5α chromosome using λ-Red recombinase-mediated gene replacement, generating the Δ*ompC* mutant strain. (**3**) Insertion of *ompC* into the pTrc vector: the pTrc expression plasmid was digested with *Sal*I and *Hind*III, and the *ompC* fragment was inserted downstream of the pTrc promoter. The resultant recombinant plasmid (pTrc + *ompC*) was transformed into the *E. coli* DH5α Δ*ompC* strain. (**4**) Insertion of mimotope and surface expression: the recombinant pTrc-*ompC* plasmid was further digested with *Pst*I to introduce mimotope sequences into the *ompC* coding sequence. Expression of the OmpC–mimotope fusion protein resulted in the surface display of mimotopes on *E. coli* DH5α Δ*ompC* cells.

The DNA was isolated from the *E. coli* DH5α strain using the Quick-DNA Fungal/Bacterial Miniprep Kit (Zymo Research, Irvine, CA, USA) according to the manufacturer’s instructions. The DNA concentration was measured using NanoDrop (Thermo Fisher Scientific). The overhang primers with *Sal*I and *Hind*III sites (forward primer 5′-CCGGTCGACATGAAAGTTAAAGTACTGTCCC-3′ and reverse primer 5′-GGGAAGCTTTTAGAACTGGTAAACCAGACC-3′) were designed to amplify *ompC* from the isolated DNA via PCR. The PCR products were sequenced (Azenta Life Sciences). For cloning, the sequenced PCR product and pTrc99a vector were double-digested with *Sal*I (New England Biolabs) and *Hind*III (New England Biolabs) and ligated using DNA ligase (New England Biolabs), according to the manufacturer’s instructions. The ligated products were transformed into the *E. coli* DH5α Δ*ompC* strain, and recombinant colonies were selected. To enable precise insertion of the *C. hepaticus* mimotope into external loop 8 of OmpC, the parental pTrc99a vector carrying *ompC* was first engineered. A short gene fragment containing unique *Not*I and *Xho*I restriction sites was introduced into the unique *Pst*I restriction site within the *ompC* coding sequence. This insertion fragment was generated by annealing two complementary oligonucleotides (Integrated DNA Technologies (IDT), Coralville, IA, USA) flanked by *Not*I and *Xho*I sites. Subsequently, the specific *C. hepaticus* mimotope sequence with a C-terminal 6xHis-tag was cloned into the newly engineered sites via directional ligation using *Not*I/*Xho*I restriction sites. The resulting recombinant plasmid was transformed into the *E. coli* DH5α Δ*ompC* strain, and positive colonies were selected for downstream analysis. The stepwise construction of the OmpC-based surface display system using the *E. coli* DH5α Δ*ompC* is illustrated in Figure 1.

### 2.7. Confirmation and Validation of Mimotope Expression in the ΔompC DH5α Strain

To confirm expression of the mimotope fusion protein in the *E. coli* DH5α Δ*ompC* strain, the bacterial culture was induced with IPTG, and the resulting product was validated by western blot analysis. The recombinant protein was assessed by isolating the OMP fraction from the induced recombinant Δ*ompC* DH5α strains using the N-lauryl sarcosine method [48]. Proteins were separated by sodium dodecyl sulfate–polyacrylamide gel electrophoresis (SDS-PAGE) and transferred to a polyvinylidene difluoride (PVDF) membrane for confirmation. The His-tagged OmpC–mimotope fusion protein was specifically detected using a Penta-His primary antibody (QIAGEN, Germantown, MD, USA) and a horseradish peroxidase (HRP)-conjugated goat anti-mouse IgG (H+L) secondary antibody (Thermo Fisher Scientific, Waltham, MA, USA), followed by staining the membrane with Pierce^TM^ 3,3′-diaminobenzidine (DAB) Substrate Kit to visualize the protein (Thermo Fisher Scientific, Waltham, MA, USA).

### 2.8. Confirmation and Validation of Mimotope Expression in the ∆aroA∆asd Double Mutant APEC PSUO78 Strain

Following in vitro confirmation of sandwich fusion mimotope expression in *E. coli* DH5α Δ*ompC*, we adopted the same strategy to express the mimotopes in the APEC PSUO78 ∆*aroA*∆*asd* double mutant strain. The *ompC* gene sequence containing the *C. hepaticus* mimotope and 6×His-tag was excised from the pTrc vector and subcloned into the pYA3342 expression vector using *Sal*I and *Hind*III restriction enzyme sites. The recombinant pYA3342 plasmid was then transformed into the APEC PSUO78 Δ*aroA*Δ*asd* double mutant strain. Successful transformation and subsequent expression of the recombinant OmpC–mimotope fusion protein within APEC PSUO78 were confirmed by western blotting. The His-tagged fusion protein was immune-detected in the outer membrane fraction of the transformants, thereby validating the suitability of the construct for use as a live-attenuated bacterial vaccine.

### 2.9. Bacterial Strains Used for Immunogenicity and Efficacy Studies

As described above, we developed the ∆*aroA* ∆*asd* double mutant *E. coli* PSUO78 strains expressing one of the 14 mimotopes of *C. hepaticus*. The specific details of the *C. hepaticus* mimotopes, including their sequences, chicken experimental groups, and the corresponding *E. coli* vaccine strain genotypes, are summarized in Table 2.

### 2.10. Experimental Animals and Housing

A total of 120 SPF embryonated white Leghorn chicken eggs were procured from AVS Bio, LLC (Norwich, CT, USA). Upon arrival, the egg surfaces were disinfected according to standard laboratory protocols. The eggs were incubated at 37.5 °C (99.5 °F) and 60% relative humidity (RH) for the first 18 days, with automated turning occurring approximately 10 times per day. On day 14 of incubation, the eggs were candled to discard non-viable embryos. On day 18, the incubator parameters were adjusted to 36.5 °C (97.7 °F) and 65% RH to facilitate hatching. Following hatch, chicks were sexed, and male chicks were culled. The remaining female chicks were leg-banded and randomly assigned to experimental groups at 4 weeks of age, with seven hens per pen. The chickens were provided ad libitum access to commercial feed and water throughout the study period.

### 2.11. Vaccination of Chickens with Mimotope-Expressing E. coli

Nine groups of seven chickens each were used. The chickens in each group were vaccinated via the oral route (oral gavage) by administering recombinant vaccine constructs using gavage needles. Blood samples were collected (pre-immune sera) prior to vaccination. Seven of the groups received a mixture of of 2 mimotope expressing ∆*aroA* ∆*asd E. coli* PSUO78 at a total dose of 10^9^ CFU in 1 mL of PBS at 16 and 18 weeks of age. Subsequently, all groups, except the unchallenged control group, were challenged with a mixture of three *C. hepaticus* strains, USA1, USA5, and USA52, by administering 10^10^ CFU/mL/bird of bacteria in Brucella broth via direct oral gavage at 26 weeks of age. The chickens were monitored daily for clinical signs and euthanized two weeks post-infection. At necropsy, macroscopic lesions were recorded, and samples (liver, spleen, bile, and cecal contents) were collected for bacterial isolation and identification using culture and PCR methods. The unchallenged negative control group received Brucella broth only. The age, route, dose of vaccination, and challenge of the vaccination and control groups are shown in Table 3.

### 2.12. Assessment of SLD Severity by Gross and Histopathological Liver Lesions

The plan to assess disease development included the evaluation of both clinical signs and gross pathology. The birds were monitored daily for clinical signs that were consistent with SLD, and disease severity was determined qualitatively. For quantitative pathology, the degree of hepatic damage was standardized using a gross liver lesion score ranging from 0 to 3, based on the macroscopic characteristics and enumeration of multifocal, small, round, and white foci on the liver surface. A score of 0 denoted the absence of lesions; a mild score of 1 was assigned for <20 foci; a moderate score of 2 represented 20 to 60 foci (>20 but <60); and a severe score of 3 was assigned for >60 foci or when they were too numerous to count (TNTC). Furthermore, any bird experiencing mortality or requiring euthanasia due to severe SLD clinical signs was automatically assigned a maximum score of 3. However, no birds developed clinical signs of SLD or gross liver lesions during the study period. The liver tissues from birds suspected of SLD were fixed in 10% neutral phosphate-buffered formalin for histopathological examination, but histopathology was not required as no gross lesions were observed [22].

### 2.13. Culture Isolation and Identification of C. hepaticus from Liver and Bile Samples

Liver and bile samples were collected for bacterial culture and identification of *C. hepaticus*. The bile samples were aseptically collected from the gallbladder, and 300 μL of each sample was spread onto Columbia agar plates supplemented with 5% sheep blood (Thermo Fisher Scientific). All primary plates were incubated at 37 °C under microaerophilic conditions (generated using a Mitsubishi^TM^ AnaeroPack-MicroAero gas generator, Thermo Fisher Scientific) for 7 days [49]. For the liver, samples were homogenized and plated directly onto Brucella agar supplemented with *Campylobacter* selective supplements I (Oxoid™ Modified Preston *Campylobacter* Selective Supplement) and II (Oxoid^TM^ Campylobacter Growth Supplement) and 5% laked horse blood (Thermo Fisher Scientific). A portion of each homogenized sample was inoculated into Brucella broth containing the same selective supplements. Both agar plates and broth cultures were incubated at 37 °C under microaerophilic conditions for 7 days. Following incubation, Brucella broth culture was subcultured onto Columbia agar plates supplemented with 5% sheep blood and incubated for an additional 7 days under the same microaerophilic conditions [15,21,50,51]. The suspected colonies from the final plates were then verified as *C. hepaticus* using an established PCR as described below. The recovery rate of *C. hepaticus* was expressed as a percentage for each experimental group. This was calculated by dividing the number of birds from which *C. hepaticus* was successfully recovered (detected via culture or PCR) by the total number of birds sampled within that specific group and multiplying it by 100.

### 2.14. C. hepaticus Identification by a PCR Targeting the Glycerol Kinase Gene

The DNA was isolated from the bacterial colonies using a Quick-DNA Fungal/Bacterial Miniprep Kit (Zymo Research) according to the manufacturer’s instructions. The isolated DNA was used to conduct a *C. hepaticus-*specific PCR targeting the glycerol kinase (*glk*) gene as previously described [52]. The PCR products were separated on a 1.5% agarose gel, and the amplicons were visualized at 463 bp and 308 bp using a GelDoc Go Gel Imaging System (Bio-Rad, Hercules, CA, USA).

### 2.15. Measurement of Epitope-Specific IgG by ELISA

This assay was performed to quantify serum IgG antibodies specific to the synthetic epitope in vaccinated chickens. The 96-well microtiter plates were initially coated with 10 μg of epitope in a carbonate–bicarbonate buffer (pH 9.4), overnight at 4 °C. The plates were then washed three times with PBS-Tween 20 (0.05%) and blocked for 1 h with the same buffer to inhibit non-specific binding. Next, 100 μL of serum diluted to 1:10 in PBS was incubated in each well for 1 h at 37 °C. Following stringent washing steps, the captured IgG was detected by adding an alkaline phosphatase (AP)-conjugated goat anti-chicken IgG (Fc fragment) secondary antibody (Thermo Fisher Scientific) and incubating for 1 h at room temperature. After three final washes, the enzyme substrate solution, p-nitrophenyl phosphate (PNPP), was added. After 30 min of incubation, the optical density (OD) of the colorimetric signal was measured at 405 nm. The final OD values were recorded as the mean of duplicate wells, and the validity of the assay was ensured using positive and negative control sera and blank wells [53,54].

### 2.16. Measurement of Epitope-Specific IgA by ELISA

This assay was performed to quantify mucosal IgA antibodies against the synthetic epitope using intestinal washings collected from the study chickens. Intestinal washings were collected during necropsy, centrifuged, and the supernatant was stored at −20 °C after stabilization with phenylmethanesulfonyl fluoride, sodium azide, and bovine serum albumin. The ELISA itself began with the coating of 96-well microtiter plates overnight at 4 °C with 10 μg of the synthetic epitope per well in a carbonate–bicarbonate buffer (pH 9.4). The plates were washed three times with PBS-Tween 20 and blocked for 1 h. Subsequently, 100 μL of processed intestinal washings, diluted to 1:10 in PBS, was added to each well and incubated for 1 h at 37 °C. After stringent washing, captured IgA was detected by adding an AP-conjugated goat anti-chicken IgA (α-chain) secondary antibody (Thermo Fisher Scientific) and incubating for 1 h at room temperature. After three final washes, the enzyme substrate solution PNPP was added and incubated for 30 min, and the resulting OD was measured at 405 nm wavelength. The OD values were recorded as the mean of duplicate wells, and positive/negative control sera and blank wells were used for quality control [53,54].

### 2.17. Bacterial Genomic DNA Extraction from Liver, Bile, and Cecal Contents for TaqMan Real-Time Quantitative PCR (TaqMan-qPCR)

The genomic DNA was extracted from liver and bile samples using the DNeasy Blood and Tissue Kit (QIAGEN). The cecal contents were subjected to genomic DNA extraction using a QIAamp PowerFecal Pro DNA Kit (QIAGEN) according to the manufacturer’s instructions. The DNA concentrations were measured using a NanoDrop Eight Spectrophotometer (Thermo Scientific).

### 2.18. Estimation of C. hepaticus Loads in the Cecal Contents, Bile and Liver Samples Using Conducting TaqMan-qPCR

The quantitative *C. hepaticus* loads in IgG- and IgA-positive vaccinated, challenged groups were calculated using TaqMan-qPCR assay, as described by Gadu et al., 2025 [55]. The TaqMan assay targeted the highly conserved *glk* gene of *C. hepaticus*. For liver samples, relative quantification was performed using the chicken *β*-actin (*ACTB*) gene as a host internal control. Conversely, for cecal contents, absolute quantification of *C. hepaticus* DNA was conducted without normalization to a host house-keeping gene. The oligonucleotide primers and probe sequences (IDT) were as follows: for ACTB, the forward primer was 5′-TCCCTGTATGCCTCTGG-3′, the reverse primer was 5′-CTACAGCTTCACCACCA-3′, and the TaqMan probe was 5′-CCACCGCAAATGCTTCTAGACCCA-3′ (labeled with FAM at the 5′ end and quencher dye at the 3′ end); for *C. hepaticus*, the forward primer was 5′-TAATGCAGCTTGTTGATCTCCG-3′, the reverse primer was 5′-ATGGGTATACAGCTACTAGATGG-3′ and the TaqMan probe was 5′-AGTGAAATTCCAATAGCAGGTATAGCCG-3′ (labeled with FAM at the 5′ end and quencher dye at the 3′ end). All reactions were performed in a 20 μL total volume containing 1× TaqMan Fast Advanced Master Mix (Applied Biosystems, Thermo Fisher Scientific), 0.4 μM of each primer, 0.2 μM probe, and 5 μL of DNA template, with nuclease-free water were added to reach the final volume. Each sample was run in triplicate using a QuantStudio 5 Real-Time PCR System (Thermo Fisher Scientific).

### 2.19. Statistical Analysis

Statistical comparisons of mean IgA and IgG antibody concentrations between the different vaccination and unvaccinated control groups were performed using GraphPad Prism version 10.0.0 (GraphPad Software, San Diego, CA, USA). For the IgG concentration comparisons, a paired t-test (for normally distributed data) or the Wilcoxon signed-rank test (for non-parametrically distributed data) was conducted. For the IgA from the intestinal washings, a one-sample *t*-test (for normally distributed data) or the Mann–Whitney U test (for non-parametrically distributed data) was used to test the hypothesis that antibody concentrations were higher in the vaccinated groups than in the unvaccinated groups. The *C. hepaticus* loads across the vaccinated challenged groups and unvaccinated challenged positive control group were compared using one-way ANOVA, and Tukey’s honestly significant difference (HSD) post hoc test was performed to compare all possible pairs of group means after obtaining a significant result from the one-way ANOVA. The statistical significance was set at *p* ≤ 0.05.

## 3. Results

### 3.1. Preparation of Heat-Killed C. hepaticus Whole-Cell Antigen and Production of Hyperimmune Sera

The hyperimmune serum that was collected two weeks after booster vaccination with heat-killed *C. hepaticus* demonstrated robust antibody production against *C. hepaticus*, with higher antibody levels than those observed in the negative control bird serum. After purification with caprylic acid, the IgG fraction from the hyperimmune serum was concentrated and quantified, yielding a concentration of 740 µg/mL.

### 3.2. Phage Display and Mimotope Identification

The plates were coated with 74.8 µg/mL polyclonal antibodies in a 0.1 M NaHCO_3_, pH 8.6 buffer to construct the phage library. The Ph.D.-12 phage display peptide library was successfully screened against the generated polyclonal antibodies via three rounds of biopanning. The selection stringency was progressively enhanced across rounds by systematically increasing the detergent Tween 20 concentration in the wash buffers and the number of wash steps, successfully enriching phages displaying peptides with a high specific affinity for the target antibodies. The total number of phage clones observed was 1 × 10^10^, 5 × 10^8^, and 3.3 × 10^11^ CFU/mL in rounds one, two, and three, respectively. Following the third round, 100 individual phage plaques were randomly isolated, amplified in *E. coli* K12 ER2738, and the supernatant was submitted for Sanger sequencing. The translation of the resulting DNA sequences identified 32 unique 12-amino-acid peptide mimotopes. BLASTp analysis was conducted using NCBI BLAST+ against the non-redundant (nr) protein database (search performed on 15 March 2022) with default parameters. Using subcellular localization predictors (Cell-PLoc and PSORTb), 14 mimotopes were ultimately selected as they exhibited homology to proteins predicted to be surface-exposed. Sequences with less than three contiguous amino acid residues of similarity were excluded to prioritize biologically relevant epitopes (Table S1).

**Figure 2:**
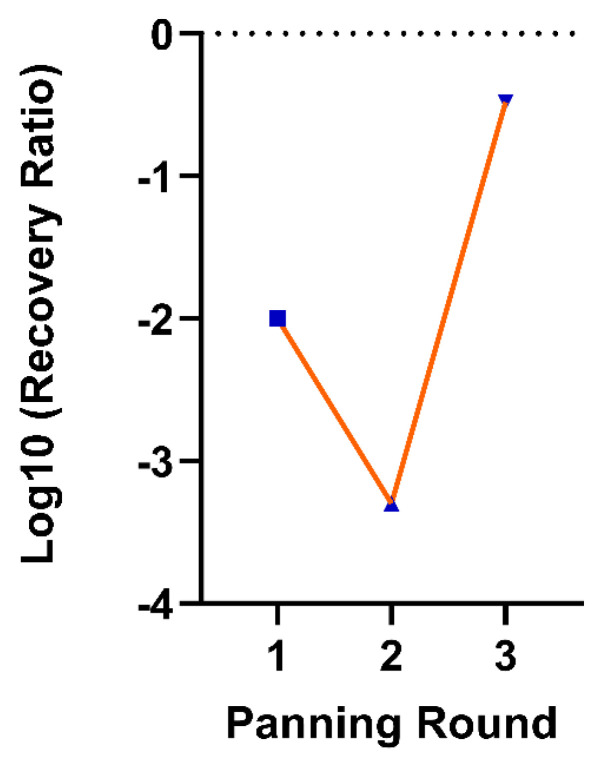
Phage display enrichment over three biopanning rounds. The y-axis represents the log_10_ recovery ratio, and the x-axis indicates the biopanning rounds. Blue squares represent the phage recovery ratio for each round. The orange line connects the data points to illustrate the trend of enrichment across successive rounds.

The efficiency of biopanning was assessed by calculating the phage recovery ratio (PFU_out_/PFU_input_) across three selection rounds. The initial phage library input (PFU_input_) was consistently maintained at 1 × 10^12^ for each round of selection. The results demonstrated significant enrichment of target-specific phages by the third round as follows: for round-1 (R1) recovery was 1 × 10^10^, yielding a recovery ratio of 1 × 10^−12^ (Log10 = −2.0); for round-2 (R2) recovery was 5 × 10^8^, yielding a recovery ratio of 5 × 10^−4^ (Log10 = −3.3); and for round-3 (R3) recovery was 3.3 × 10^11^, yielding a recovery ratio of 3.3 × 10^−1^ (Log10 = −0.48). Figure 2 shows the round-wise recovery rates of phages during the phage display.

### 3.3. Multiple Sequence Alignment and Epitope Consensus Analysis

**Figure 3:**
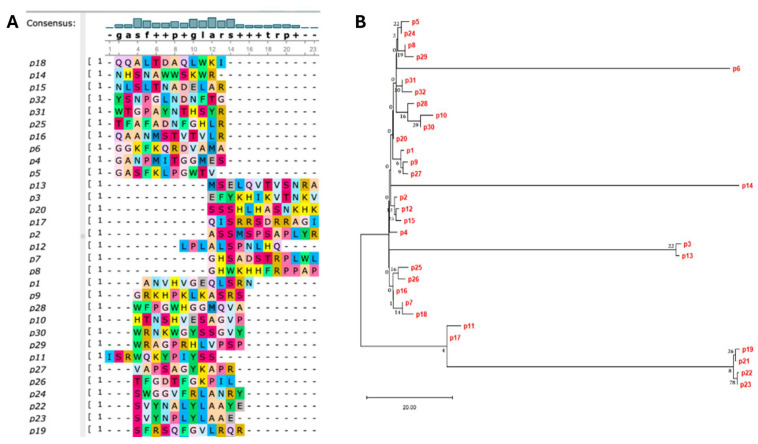
Molecular analysis of phage-displayed mimotopes. (**A**) Multiple sequence alignment of the 32 unique mimotopes (p1–p32) recovered after selection against polyclonal antibodies. The sequences were aligned using Clustal Omega and visualized using UniProUGENE. Residues are color-coded by amino acid identity to facilitate visualization of sequence similarity and variation across clones. The degree of residue conservation is displayed in the histogram and the consensus sequence above the 23 aligned positions. The key conserved motifs G-A-S-F (positions 2–5), G-L-A-R-S (positions 10–14), and T-R-P (positions 18–20) represent the consensus epitope recognized by the polyclonal antibody pool. (**B**) Maximum likelihood (ML) phylogenetic tree showing the evolutionary relationships among 32 mimotope sequences. The branch lengths are proportional to the number of substitutions per site, as indicated by the scale bars. The numbers at the nodes represent the bootstrap support values (out of 1000 replicates). The tree was rooted using the designated outgroup sequence.

The multiple sequence alignment (MSA) of the 32 selected mimotope peptide sequences (p1–p32) was generated using Clustal Omega (https://www.ebi.ac.uk/jdispatcher/msa/clustalo Version 1.2.4. on 9 July 2023) and visualized with UniproUGENE (https://ugene.net/) (Figure 3A), spanning 23 amino acid residues (positions 1–23). The analysis revealed a conserved core-aligned region spanning positions 2–20.

#### Identification of Conserved Motifs

The MSA identified three major blocks of high conservation within the mimotope sequences. The N-terminal motif (G-A-S-F): A highly conserved block, G-A-S-F (glycine-alanine–serine–phenylalanine), is present at alignment positions 2 through 5. The central Motif (G-L-A-R-S): The motif G-L-A-R-S (glycine–leucine–alanine–arginine–serine) spans positions 10 through 14. The C-Terminal Motif (T-R-P): The motif T-R-P (threonine–arginine–proline) is found at positions 18–20. The consensus sequence demonstrated variability in the segments linking the conserved motifs, suggesting a flexible structure or function. This variability was noted at several positions: positions 6, 7, 8, 9, 15, 16, and 17, which are marked with low-conservation symbols (‘+’ or ‘p’) in the consensus sequence. Furthermore, alignment boundaries with the absence of a strong consensus call (blank space) at positions 1, 22, and 23 were observed.

### 3.4. Phylogenetic Analysis of Mimotopes

The genetic relationships among the 32 unique mimotopes (p1 to p32) recovered from phage display biopanning and downstream sequencing were analyzed using the maximum likelihood (ML) method. A phylogenetic tree was constructed based on multiple sequence alignments and rooted with a designated outgroup sequence. The reliability of the inferred topology was evaluated using 1000 bootstrap replicates. The resulting ML tree (Figure 3B) revealed that the amino acid sequences of mimotopes segregated into five primary, distinct evolutionary clades (A–E), indicating considerable sequence diversity among the selected peptides. The average branch length suggests a moderate level of substitution per site, reflecting both conserved and divergent sequence features in the dataset used.

Clade A emerged as the dominant and most highly supported cluster, comprising 13 mimotopes (p25, p26, p16, p7, p18, p11, p17, p3, p13, p19, p21, p22, and p23), supported by a strong bootstrap value of 98%. The tight clustering within Clade A suggests that these mimotopes share a conserved binding motif, likely representing the principal epitope recognized by the target antibodies. Clade B included nine mimotopes (p31, p32, p28, p10, p30, p20, p1, p9, and p27) and displayed a bootstrap value of 82%, indicating a moderately conserved group. The sequence diversity within this cluster suggests the potential recognition of a related but distinct antigenic surface or a conformational epitope. Clade C comprised four closely related mimotopes (p5, p24, p8, and p29), supported by a bootstrap value of 76%. The relatively short internal branches within this cluster suggest minimal divergence, possibly reflecting a set of mimotopes derived from a common structural mimic of the same antigenic determinant. Two singleton branches, Clade D (p14) and Clade E (p6), were positioned distantly from the main clusters, each supported by high bootstrap values (>90%), indicating a strong individual lineage separation. The longer branch lengths for these mimotopes imply substantial sequence divergence, possibly representing unique or low-affinity binding motifs that are distinct from those of the dominant clades.

Overall, the phylogenetic analysis confirmed the presence of multiple distinct mimotope clusters with varying degrees of sequence conservation. The dominance of Clade A, along with the presence of several divergent clades, underscores the heterogeneity of the antibody-binding motifs recovered during biopanning.

### 3.5. Creation and Validation of Recombinant E. coli ΔompC Strain Expressing C. hepaticus Mimotopes

#### 3.5.1. Creation of the *E. coli* DH5α ∆*ompC* Mutant Strain

The *ompC* gene in *E. coli* DH5α was successfully inactivated using the λ Red recombinase system. The correct gene replacement was confirmed by a PCR, which showed that the *ompC* locus was replaced with the CmR cassette. The curing of pKD46 and the subsequent FLP-mediated excision of the CmR marker using pCP20 was successful, generating a marker-free *E. coli* DH5α Δ*ompC* strain, as validated by a PCR and the loss of resistance markers.

#### 3.5.2. Confirmation of Mimotope Expression in *E. coli* DH5α Strain

**Figure 4:**
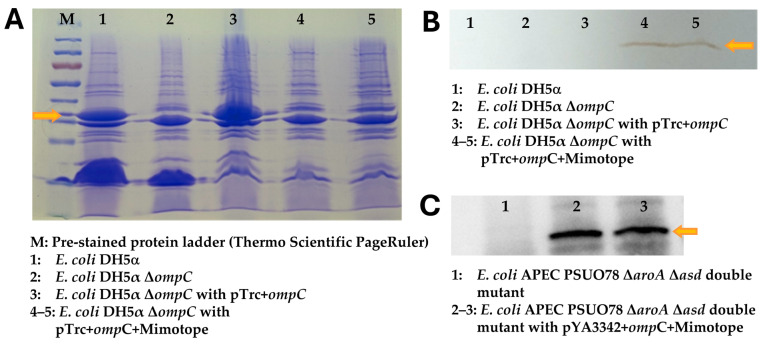
Expression and confirmation of the surface display of *C. hepaticus* mimotopes as a fusion with OmpC in *E. coli* DH5α Δ*ompC* and APEC PSUO78 Δ*aroA* Δ*asd* double mutant strains. (**A**) Coomassie brilliant blue–stained gel picture showing the outer membrane proteins extracted from the recombinant *E. coli* DH5α Δ*ompC* strains. The arrow indicates the position of OmpC (30 KDa). (**B**) Western blot image showing the recombinant His-tagged OmpC protein extracted from the *E. coli* DH5α Δ*ompC* strain. The arrow indicates the 48 kDa OmpC + mimotope fusion (lanes 4 and 5 in (**A**)). (**C**) The western blot showing the recombinant outer membrane protein fractions extracted from APEC PSUO78 Δ*aroA* Δ*asd* carrying three different mimotopes. The arrow indicates the 48 kDa OmpC+ mimotope.

The *ompC* gene was engineered to contain *Not*I and *Xho*I sites for the insertion of the mimotope-His-tag cassette. The double-stranded *Not*I/*Xho*I insert encoding the mimotope was successfully cloned into the pTrc99a-*ompC* vector. The transformation into the Δ*ompC* DH5α strain was confirmed by plasmid analysis. The construction and confirmation of *ompC* mutant and recombinant *E. coli* strains are described in Figure S1 using agarose gel pictures from A-H. The western blot analysis of the OMP fraction from IPTG-induced Δ*ompC* DH5α pTrc99a-OmpC-mimotope confirmed the expression of fusion proteins. The anti-His tag primary antibody detected a clear band at the predicted molecular weight of 48 kDa (Figure 4A,B), confirming the successful translation and outer membrane localization of the OmpC–mimotope-His fusion protein in *E. coli*.

### 3.6. Validation of Mimotope Expression in APEC PSUO78 ΔaroAΔasd Strain

The *ompC*-mimotope-6xHis construct was successfully subcloned into the pYA3342 vector using *Sal*I and *Hind*III sites. The resulting expression vector was transformed into the live-attenuated vaccine vector strain APEC PSUO78 Δ*aroA* Δ*asd*. Western blot analysis of the OMP fraction from APEC PSUO78 transformants confirmed the expression of the His-tagged fusion protein containing *C. hepaticus* mimotopes (Figure 4C). A band corresponding to the expected molecular weight of 48 kDa was clearly detected by the anti-His antibody, demonstrating successful surface display of the *C. hepaticus* mimotope on the APEC PSUO78 Δ*aroA* Δ*asd* live-attenuated strain.

### 3.7. Protection Efficacy Against C. hepaticus Challenge

All chickens were euthanized two weeks post-challenge. At the time of necropsy, all chickens appeared normal with no observable signs of illness. No gross pathological changes were observed on the surface of the livers or other internal organs. Liver and bile samples were collected for *C. hepaticus* culture and isolation. The chickens in five vaccinated groups (groups-1, -2, -4, -6, and -7) showed 100% protection against *C. hepaticus* challenge, as demonstrated by the absence of *C. hepaticus* recovery in liver and bile samples, whereas the unvaccinated challenged group showed 100% recovery of *C. hepaticus*. Recovered bacteria were confirmed as as *C. hepaticus* by a conventional PCR (Figure S2). Table 4 shows *C. hepaticus* recovery and vaccine protection rates for all mimotope-vaccinated and positive control groups.

### 3.8. Immunogenicity of Mimotope-Expressing ∆aroA ∆asd APEC PSUO78 Strains

#### 3.8.1. Serum IgG Responses

**Figure 5:**
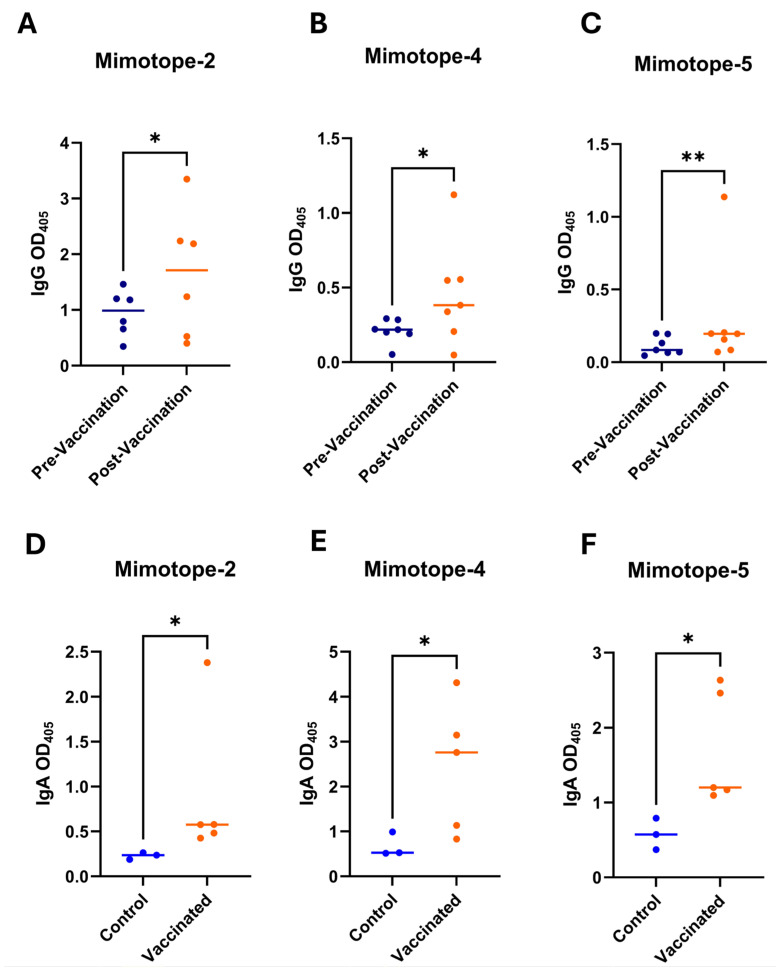
The IgG and IgA antibody responses induced by mimotope-2 (FliK), mimotope-4 (FLAA) and mimotope-5 (MOMP) expressing APEC PSUO78 ∆*aroA* ∆*asd* double mutant strain. (**A**–**C**) serum IgG antibody responses induced by mimotope expressing ∆*aroA* ∆*asd* double mutant *E. coli* PSUO78 (pre-vaccination: the serum samples were collected before vaccination; post-vaccination: the serum samples collected after booster vaccination) and (**D**–**F**) mucosal IgA antibody responses induced by mimotope expressing ∆*aroA* ∆*asd* double mutant *E. coli* PSUO78. * *p* ≤ 0.05 (significant) and ** *p* ≤ 0.01 (moderately/highly significant). (Control: intestinal washings collected from unvaccinated–unchallenged birds; vaccinated: intestinal washings collected from the vaccinated birds.).

Following the two-dose oral vaccination regimen, a significant difference in antigen-specific systemic antibody response (IgG) was observed between the vaccinated groups and the control unvaccinated groups. Among the seven vaccinated groups, three groups (groups-4, -6, and -7) showed elevated IgG serum levels specific to three mimotopes (mimotopes-2, -4, and -5). Among the three mimotopes that showed promising results, two contained flagellar components (flagellar hook-length control protein FliK and flagellin A FlaA), and the other contained a major outer membrane protein (MOMP) sequence. The OD values for the mimotope-2 (FliK)-vaccinated group were normally distributed, and a paired t-test was conducted, which demonstrated a statistically significant increase in post-vaccination IgG OD values compared to the pre-vaccination values. For the mimotope-4 (FlaA) and mimotope-5 (MOMP) vaccinated groups, the data were not normally distributed; thus, the Wilcoxon matched pairs signed rank test was performed. The mimotope-4 and mimotope-5 vaccinated groups showed a statistically significant increase in the IgG OD values post-vaccination. A summary of the statistics performed to measure the IgG responses for mimotopes-2, -4, and -5 is summarized in Table S2, while the serum IgG antibody responses are presented in Figure 5.

#### 3.8.2. Serum IgA Responses

To assess mucosal immune responses against *C. hepaticus* mimotope vaccination, mucosal IgA levels were measured in intestinal washings that were collected during necropsy. The IgA responses were evaluated in three vaccinated groups (groups-4, -6, and -7), which showed no *C. hepaticus* recovery from the liver and bile and showed positive IgG responses. All three groups exhibited increased antigen-specific IgA levels compared to unvaccinated, unchallenged control birds. Figure 4 shows the IgA responses induced by the corresponding mimotope-expressing ∆*aroA* ∆*asd* APEC PSUO78 strains. IgA levels were quantified as OD values at 405 nm (OD_405_). The data from the three vaccinated groups (mimotopes-2, -4, and -5) and the unvaccinated control group were not normally distributed; therefore, the Mann–Whitney test was used to compare IgA levels between the vaccinated and control groups. A summary of the statistical analyses of mucosal IgA responses is provided in Table S3 and Figure 5.

### 3.9. Estimation of C. hepaticus DNA Loads in the Liver and Cecal Contents of the IgG- and IgA-Positive Vaccinated Challenged Groups and Unvaccinated Challenged Positive Control Group

TaqMan-qPCR was used to measure and compare the *C. hepaticus* DNA loads in the liver and cecal contents of the vaccinated and positive control groups. To assess the technical quality of the assay, we first established standard curves for the absolute quantification of both the host (*ACTB* gene) and *C. hepaticus* (*glk* gene) DNA (Figure S3). The correlation coefficient values for *glk* and *ACTB* genes were very reliable at 0.999 and 0.997, respectively. The amplification efficiencies for *glk* and *ACTB* genes were detected as 96.3% and 82.5%, respectively. The *C. hepaticus* load was calculated as the ratio of the *C. hepaticus* genome to the host genome.

For the glycerol kinase gene: CT = −3.413 × log_10_ (copies) + 41.735.

For the beta-actin gene: CT = −3.875 × log_10_ (copies) + 45.698.

### 3.10. C. hepaticus DNA Loads in Liver Samples

**Figure 6:**
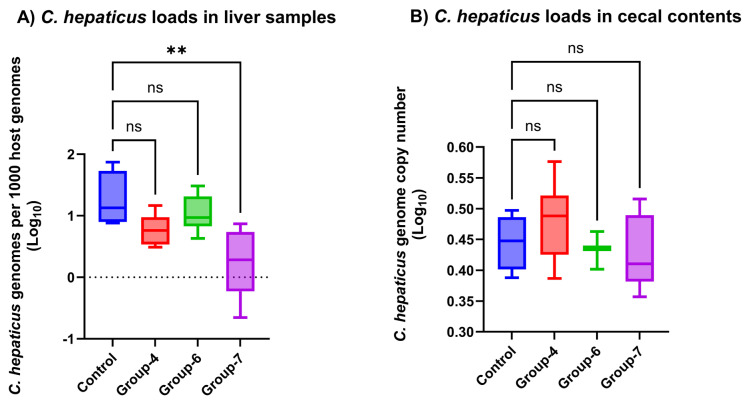
Comparison of *C. hepaticus* loads in liver and cecal samples across the experimental groups by quantitative real-time PCR. Group-4 (Mimotope-2, FliK), group-6 (mimotope-4, flagellin A), group-7 (mimotope-5, MOMP), and the control group (positive control: unvaccinated-challenged control group). ns: *p* > 0.05 (not significant), and **: *p* ≤ 0.01 (moderately/highly significant).

A one-way ANOVA was conducted on the log_10_-transformed normalized bacterial load to compare the four experimental groups (vaccinated and positive control groups). The analysis revealed a statistically significant difference in the mean *C. hepaticus* load across the groups: the F (3, 16) = 5.230, and the *p*-value is 0.0105. The HSD post hoc test revealed that group-7 showed a statistically significant reduction in *C. hepaticus* load compared to the positive control group. The descriptive statistics, one-way ANOVA, and post hoc test results are mentioned in Tables S4 and S5, while the distribution of *C. hepaticus* loads across treatment groups is visualized in Figure 6A.

### 3.11. C. hepaticus DNA Loads in Cecal Contents

The absolute *C. hepaticus* genome counts from the cecal contents were transformed into log_10_ prior to the analysis. A one-way ANOVA of the three vaccinated groups (groups-4, -6, and -7) and positive control group revealed no significant difference in the log_10_ absolute *C. hepaticus* loads across the groups: the F (3, 16) = 1.001, and the *p*-value = 0.4180. The descriptive statistics of the absolute *C. hepaticus* load (mean, log_10_, load) in the cecal contents between the vaccinated and control groups are shown in Table S6, while the distribution of *C. hepaticus* loads across the experimental groups is shown in Figure 6B.

## 4. Discussion

In this study, we developed a new live-attenuated avian *E. coli* strain engineered to express *C. hepaticus* mimotopes on its surface, which was intended for use as a dual-purpose vaccine against SLD and colibacillosis in poultry. This approach integrates a phage display method for mimotope identification with advanced genetic engineering to incorporate the selected mimotopes into a live-attenuated *E. coli* vector, resulting in a promising platform for the prevention of SLD and colibacillosis, including *E. coli* egg yolk peritonitis in layer chickens. The successful development of a novel live-attenuated APEC vaccine to deliver *C. hepaticus* mimotopes was supported by three major outcomes of this study: (i) the identification of high-binding affinity immunogenic mimotopes of *C. hepaticus*, (ii) the characterization of conserved G−A−S−F/G−L−A−R−S/T−R−P consensus motifs, and (iii) the confirmation of the expression of *C. hepaticus* mimotopes on the surface of the APEC PSUO78 Δ*aroA* Δ*asd* strain as an OmpC–mimotope fusion. Although vaccine efficacy studies are still in progress, the surface display system described here represents a robust and adaptable *E. coli* platform for displaying heterologous mimotopes and other small peptides, enabling a wide range of applications in antigen discovery and vaccine development.

In typical phage-display selections, most of the initial output phage libraries are either non-binding or weakly bound and are removed during the initial washing steps [56]. To further improve the selection, we added an additional negative selection step before the first round of panning by incubating the phage library with polyclonal sera from the negative control birds to eliminate unwanted binding and non-specific phages that simply stick to the non-specific areas of the coated plate [57]. During the second round of panning, we noticed a drastic reduction in phage recovery, which might be due to the use of higher concentrations of detergent (Tween-20) in the wash buffer and the removal of weak or non-specific phages [58]. The remaining phage population was enriched for clones that specifically recognized the target molecules. The phage recovery ratio increased by nearly three orders of magnitude between R2 and R3 (a 660-fold increase). The small pool of phages with specific binding was selectively amplified in *E. coli*, favoring the propagation of high-affinity phage clones. This resulted in a substantial increase from R2 to R3, confirming the successful enrichment and selection of phage clones displaying high affinity mimotopes of the target antigen and indicating a positive selection for specific binding clones in the final output pool [57].

The analysis of the mimotope sequences recovered by the phage display against polyclonal antibodies yielded a distinct epitope consensus, confirming the selection pressure exerted by the host immune response [29]. The MSA revealed three highly conserved motifs that are likely to form the binding interface recognized by the dominant antibody clones. The N-terminal G-A-S-F motif (positions 2–5) and C-terminal T-R-P motif (positions 18–20) represent critical conserved features. The structural importance of a constrained motif, such as T-R-P, suggests that it mimics a turn or constrained element typical of linear and conformational epitopes, a phenomenon also observed in studies mapping antigenic epitopes using mimotopes [59]. One of the major challenges in vaccinology is the design of linear peptides that successfully mimic the shape of complex conformational epitopes, which are formed by amino acids brought together through protein folding, rather than linear amino acid sequences [60]. The enrichment and selection of the T-R-P motif indicated that the linear mimotope effectively overcame this challenge by successfully mimicking the turn or loop structure of the native *C. hepaticus* protein. Furthermore, the G-L-A-R-S motif (positions 10–14) stands out for its conserved arginine (R) residue, implying that electrostatic interactions predominate in the antibody–paratope contact [61]. The electrostatic interactions are highly advantageous in vaccine design because they imply a strong, directed binding force and are typically associated with functionally critical domains of native antigens. The high variability in linker positions (6–9, 15–17) reflects the polyclonal nature of the serum, where lower-affinity clones allow substitutions in less critical sites [62]. This pattern of conserved hotspots amid variable coldspots is a known signature of consensus sequences derived from polyclonal antibody selection [63]. The extensive divergence in the N- and C-terminal extensions (positions 1, 22, and 23) further supports that antibody binding centers primarily on the core 2–20 domain, defining the minimal epitope footprint [29]. These observations jointly define G-A-S-F/G-L-A-R-S/T-R-P as the immunogenic signature motif, a promising target for the diagnostic or vaccine design for *C. hepaticus*-mediated SLD [64,65]. The MSA and phylogenetic analyses revealed a high level of concordance, supporting the reliability of the observed clustering patterns. The ClustalOmega-generated alignment revealed that mimotopes sharing conserved motifs or consensus residues were clustered closely together in the phylogenetic tree, indicating that sequence similarity was translated into evolutionary relatedness. Furthermore, verification using the sequence identity matrix confirmed that the pairwise identity values corresponded well with the branch distances of the tree. This consistency between the numerical similarity scores and tree topology suggests that both analyses capture the same underlying relationships among the mimotopes, reinforcing the robustness of the inferred sequence clustering and validating the representativeness of selected mimotopes.

A key achievement of this study was the successful engineering of a live-attenuated APEC vector for the surface expression of the selected mimotopes. We first established a cloning and expression system in *E. coli* DH5α Δ*ompC*. The deletion of the native *ompC* gene in *E. coli* DH5α using the λ Red recombinase system was necessary to ensure that the subsequent expression of the recombinant OmpC–mimotope fusion protein represented the sole source of OmpC, thereby minimizing competitive immune responses against the wild-type protein. In addition, the size differentiation of wild-type OmpC from the OmpC–mimotope-6xHis tag fusion protein was challenging due to the protein size differences between the native OmpC and the fusion protein. SDS-PAGE and western blotting with anti-His antibodies confirmed the expression of the mimotope on the surface of the *E. coli* DH5α Δ*ompC* strain. The subsequent success in subcloning and expressing the fusion protein in the APEC PSU O78 Δ*aroA* Δ*asd* double mutant strain was a defining step of this study. The subsequent success in subcloning and expressing the fusion protein in the *E. coli* Δ*aroA* Δ*asd* double mutant strain was also a defining step of this study. The Δ*aroA* mutation attenuates the bacterium by rendering it auxotrophic for aromatic amino acids and unable to survive in a nutrient-limited host environment [66]. Similarly, *asd* deletion creates an absolute auxotrophy for DAP, a component critical for bacterial cell wall synthesis [67]. Since DAP is absent in animal tissues, the vaccine strain solely depends on the pYA3342 vector, and the strain undergoes programmed lysis shortly after administration, allowing for a transient, high-level expression of the vaccine antigen before its clearance. This transient presence is key, as the intracellular release of antigens and bacterial components in situ at the mucosal surface is highly effective in stimulating robust T cell-mediated immunity and mucosal IgA production, offering superior protection compared to traditional killed vaccines [68]. Finally, the western blot showing the attenuated APEC vaccine strain expressing mimotopes validated the safety-by-design of the construct and its readiness for in vivo assessment.

Immunogenicity and efficacy studies demonstrated that a live-attenuated APEC vaccine expressing *C. hepaticus* mimotopes can effectively protect chickens against SLD. Among the 14 tested *C. hepaticus* mimotopes, those mimicking certain epitope regions of flagellar and MOMP showed immunogenicity and protection against the *C. hepaticus* challenge. The observed elevated systemic (IgG) and local (IgA) immune responses, coupled with the protection against challenged *C. hepaticus* colonization, indicate that these mimotope antigens are promising vaccine candidates for controlling SLD.

The vaccination strategy utilizing mimotopes 2, 4, and 5 triggered a bimodal immune response that mimics the natural infection route of *C. hepaticus*. By producing both systemic IgG and mucosal IgA, the vaccine establishes a multi-layered defense. The antigen-specific IgG (seen in Figure 5A–C) serves as the primary response for tissue-level protection. Since *C. hepaticus* is a flagellated bacterium that migrates from the intestinal tract to the liver via the portal vein, these antibodies likely tag the bacteria for destruction by hepatic macrophages through opsonization, which directly correlates with the significant drop in the number of bacterial genome copies observed in the liver tissue (Figure 5A).

Simultaneously, the high titers of antigen-specific IgA (Figure 5D–F) address the initial stage of infection within the gut lumen. In poultry, secretory IgA is vital for “immune exclusion,” a process where antibodies physically coat the pathogen to block its adherence to the cecal epithelium. Interestingly, our data shows that while cecal bacterial counts remained present (Figure 6B), the systemic infection was successfully reduced. This suggests that the induced IgA functions primarily as a functional barrier, limiting the translocation of *C. hepaticus* across the gut–blood barrier rather than achieving complete bactericidal clearance in the cecal contents. Consequently, the vaccine provides “clinical protection” by preventing hepatic colonization and further preventing the resulting necrotic lesions typical of SLD, effectively decoupling the intestinal presence from the systemic pathology.

Despite the translocation mechanism of *C. hepaticus* from the gut to the liver remaining unclear, it is hypothesized that a disruption of the gastrointestinal (GI) epithelial barrier may allow *C. hepaticus* to enter the systemic circulation, migrate to the liver, and cause a clinical disease characterized by multiple necrotic foci on the liver surface. The bacteria may then reach the gallbladder/bile, from which they are shed back to the small intestine and transmitted via the fecal–oral route. The flagella play a major role in this translocation process [52,69]. Interestingly, recent transcriptomic studies on *C. hepaticus* recovered from the bile and liver showed a downregulation of the major flagellum protein *flaA*, representing a major adaptation for immune evasion [51]. This downregulation suggests that the flagellum plays a crucial role during the early stage of translocation but is later suppressed, likely as an energy-conserving mechanism or to evade host immune surveillance within the biliary niche. In the present study, vaccination with a mimotope (mimotope-4) derived from FlaA elicited strong antigen-specific IgG and IgA responses and achieved 100% protection from *C. hepaticus* colonization, demonstrating that FlaA is an effective and highly promising vaccine target.

Another important flagellar protein, FliK, is involved in the control of flagellar hook length during flagellum assembly [70,71]. Immunization with the FliK protein in mice triggered an antibody-mediated immune response and reduced mortality against a *Salmonella* challenge [72]. Although in vivo studies evaluating FliK as a vaccine antigen against *Campylobacter* spp. are lacking, reverse vaccinology approaches have identified it as a promising vaccine candidate for bovine genital campylobacteriosis (BGC) [73]. FliK is associated with the assembly of the flagellar secretory apparatus and plays a crucial role in *Campylobacter* colonization in the chicken gut [74]. In the present study, a mimotope (mimotope-2) derived from FliK induced strong antigen-specific antibody responses and conferred 100% protection against *C. hepaticus* colonization in chickens.

The outer membrane proteins (OMPs) of Gram-negative bacteria play a major role in pathogenesis, including invasion and adherence [75,76]. The OMP profile of *Campylobacter* spp. is characterized by the presence of a single major protein called MOMP [77,78]. The OMPs have been explored as vaccine candidates against *Campylobacter* [79,80]. Although MOMP is identified as immunogenic, the antibody response to MOMP is predominantly directed against conformational epitopes, which may limit the use of recombinant MOMP as a vaccine [79]. A logical approach to overcome this limitation is to use mimotopes, which structurally resemble conformational epitopes and successfully elicit protective antibody responses. In this study, the MOMP-derived mimotope (mimotope-5) elicited strong systemic and mucosal immune responses, conferring protection against *C. hepaticus* following challenge.

Although bacterial recovery from target organs by culture is the gold standard for measuring pathogen clearance, quantifying bacterial DNA in these organs can be used as an indirect measurement of pathogen clearance. Although previous studies evaluating *C. hepaticus* vaccine efficacy have not applied qPCR to measure bacterial loads in target tissues (e.g., liver and cecum), this technique is widely regarded as an acceptable approach for assessing the pathogen burden in comparable vaccine studies, such as those involving *C. jejuni* [81,82,83]. A quantitative PCR analysis of liver samples demonstrated that all three mimotope vaccine formulations (groups-4, -6, and -7) reduced the systemic pathogen burden in the liver compared to the challenged control group. Groups-4, -6, and -7 achieved *C. hepaticus* load reductions of 3.14-fold, 1.65-fold, and 9.83-fold, respectively, relative to the unvaccinated challenged control. The highest level of protection was observed in the mimotope-4 vaccine group, which resulted in a statistically significant 9.83-fold (approximately 1-log) reduction in the *C. hepaticus* load in the liver. This robust reduction in liver colonization, a key indicator of systemic dissemination, strongly supports the efficacy of the tested mimotope vaccines. Furthermore, the correlation between the observed reductions in *C. hepaticus* DNA loads, as measured by TaqMan-qPCR, and *C. hepaticus* recovery from the liver, as documented by the culture methods, strengthens the conclusion that the three selected mimotope vaccines are effective in limiting *C. hepaticus* persistence in the target tissues.

In contrast to the significant reduction observed in the liver, quantitative analysis of cecal contents using TaqMan-qPCR indicated that vaccination had no significant effect on pathogen colonization in the cecum. The *C. hepaticus* loads (log_10_ *C. hepaticus* loads) in all of the vaccinated groups remained comparable to those in the control group, with only negligible fold changes (ranging from a 1.08-fold increase to a 1.04-fold reduction). The absolute *C. hepaticus* loads detected in the cecal contents may reflect a rapid translocation of the organism from the gut to the systemic sites, resulting in an underestimation of its true abundance in the cecum. Additionally, TaqMan-qPCR quantified the DNA from both viable and non-viable bacteria. Thus, it is possible that the vaccines effectively eliminated *C. hepaticus* in the cecum, but residual DNA from killed organisms remained detectable. If so, the actual reduction in the viable bacterial burden may be substantially greater than the minimal change observed by the total DNA-based TaqMan-qPCR measurements.

The protection levels reported in this study exceeded the previous vaccine approaches explored for SLD, such as autogenous bacterins and *Salmonella*-delivered *Campylobacter* N-glycan heptasaccharide, which, to date, have demonstrated only partial protection or have primarily described immunological responses without full protective efficacy [28,84]. The enhanced efficacy observed in this study can be partially explained by the live-attenuated vector delivery system. The use of the ∆*aroA* ∆*asd* APEC strain as the vaccine vector to deliver *C. hepaticus* mimotopes ensures the delivery of the antigens via the natural infection pathways, stimulating both mucosal and systemic immune responses, which are essential for containing pathogens like *C. hepaticus* that colonize and disseminate through the GI tract [85,86,87]. Altogether, these findings support the use of mimotope-based live-attenuated bacterial vectored vaccines as a next-generation approach for controlling SLD.

Despite the successful in vitro validation of the OmpC–mimotope-6 x His fusion, this study had several limitations. First, our approach for sequence analysis relied completely on the Sanger sequencing of a subset of clones (*n* = 100) from the final round (R3) to establish a consensus motif. While this successfully identified the dominant G−A−S−F/G−L−A−R−S/T−R−P signatures, it only captured the most highly amplified sequence. A more comprehensive approach, utilizing next-generation sequencing (NGS) on the total R3 output pool, would significantly improve this method. NGS can analyze hundreds of thousands of sequences, providing a census of the final population and allowing for a more accurate, statistically robust definition of the consensus motif, potentially revealing secondary immunodominant epitopes or low-frequency high-affinity clones that were missed by random sampling [88,89,90]. The bacterial isolation and identification methods successfully isolated *C. hepaticus* from the bile and liver samples in this study, the clinical SLD could not be reproduced in unvaccinated chickens challenged with *C. hepaticus*. Recent studies have demonstrated that repeated dosing of *C. hepaticus* can induce clinical SLD (mild to moderate multifocal liver lesions) [22], though this outcome has not been consistently reproducible [84]. In addition, the repeated exposure to *C. hepaticus* to induce clinical disease may confound the assessment of vaccine efficacy, as subsequent challenge doses could function similarly to booster immunizations with a live organism. Therefore, it is difficult to distinguish whether the observed protection is attributable to the vaccine itself or to the immune priming from the repeated exposure to the challenged bacteria. Thus, the vaccine efficacy in this study was assessed based on the reduction in *C. hepaticus* colonization of the liver and bile. Three of the mimotopes (FliK, FlaA, and MOMP) examined in this study showed 100% protection against *C. hepaticus* colonization; however, the sample size was limited, and further large-scale studies are needed to confirm dose optimization and field applicability. Additionally, as reported, a portion of the vaccine efficacy may be attributed to the Δ*aroA* Δ*asd* APEC vector strain itself. The absence of a vector-only control group in this trial limits the ability to fully distinguish the vector effects from the mimotope-induced immunity and protection.

## 5. Conclusions

In summary, we engineered a rationally designed, genetically stable live-attenuated *E. coli* vector vaccine against SLD by identifying protective mimotopes and displaying them on the surface of the APEC PSUO78 Δ*aroA* Δ*asd* strain. The immunogenicity and efficacy studies using controlled challenge experiments demonstrated that three out of the 14 mimotopes conferred immunogenicity and protection against *C. hepaticus* colonization.

## Figures and Tables

**Table 1:** The bacterial strains and plasmids that were used in this study.

Strain/Plasmid	Features	Growth Conditions	Source/References
*E. coli* DH5α	*E. coli* K-12 strain (*fhuA2*Δ(*argF-lacZ*)*U169 phoA glnV44 Φ80*Δ(*lacZ*)*M15 gyrA96 recA1 relA1 endA1 thi-1 hsdR17*)	LB broth/agar at 37 °C	Thermo Fisher Scientific, Waltham, MA, USA
*C. hepaticus* strain FL2019SK1	Field strains	Brucella agar supplemented with 5% laked horse blood and *Campylobacter* supplements at 37 °C microaerophilic conditions for 5–7 days	[17]
*C. hepaticus* strains USA1, USA5 and USA52			[16,43]
APEC PSUO78	Field strain of avian pathogenic *E. coli*	LB broth/agar at 37 °C	This study (NCBI accession # CP012112.1)
APEC PSUO78 ∆*aroA* ∆*asd* double mutant strain	This study	LB broth/agar with 50 µg DAP at 37 °C	This study
*E. coli* K12 ER2738	*F’proA + B + lacIq Δ(lacZ)M15 zzf::Tn10(TetR)*/*fhuA2 glnV Δ(lac-proAB) thi-1 Δ(hsdS-mcrB)5*	LB broth/agar with 20 µg/mL tetracycline at 37 °C	New England Biolabs, Ipswich, MA, USA
pTrc99a	High copy number bacterial expression vector with a *trc* promoter	LB broth/agar with 100 µg/mL carbenicillin at 37 °C	Creative Biogene, Shirley, New York, NY, USA
pYA3342 vector	Balanced–lethal, *asd*^+^ expression plasmid	LB broth/agar without any supplements at 37 °C	[44,45]

LB broth/agar: Luria–Bertani broth or agar; DAP: diaminopimelic acid.

**Table 2:** The characterization of *C. hepaticus* mimotopes and the *E. coli* vaccine strains used in this study.

Mimotope ID	Mimotope Sequence	Group Details	Strain Details
Mimotope-1 (Hypothetical protein)	EFYKHIKVTNKV	2	∆*aroA* ∆*asd* strains with mimotope-1 in pYA3342 vector
Mimotope-2 (FliK)	GHWKHHFRPPAP	4	∆*aroA* ∆*asd* strains with mimotope-2 in pYA3342 vector
Mimotope-3 (FlgE)	ISRWQKYPIYSS	7	∆*aroA* ∆*asd* strains with mimotope-3 in pYA3342 vector
Mimotope-4 (Flagellin A)	MSELQVTVSNRA	7	∆*aroA* ∆*asd* strains with mimotope-4 in pYA3342 vector
Mimotope-5 (MOMP)	NHSNAWWSKWGR	6	∆*aroA* ∆*asd* strains with mimotope-5 in pYA3342 vector
Mimotope-6 (Hypothetical protein)	NLSLTNADELAR	5	∆*aroA* ∆*asd* strains with mimotope-6 in pYA3342 vector
Mimotope-7 (Hemagglutinin)	SSSHLHASNKHK	5	∆*aroA* ∆*asd* strains with mimotope-7 in pYA3342 vector
Mimotope-8 (FlgB)	SVRNQLGGGRGE	4	∆*aroA* ∆*asd* strains with mimotope-8 in pYA3342 vector
Mimotope-9 (CadF)	SWGGVFRLANRY	1	∆*aroA* ∆*asd* strains with mimotope-9 in pYA3342 vector
Mimotope-10 (MOMP)	TFAFADNFGHLR	1	∆*aroA* ∆*asd* strains with mimotope-10 in pYA3342 vector
Mimotope-11 (FliK)	WRAGPRHLVPSP	3	∆*aroA* ∆*asd* strains with mimotope-11 in pYA3342 vector
Mimotope-12 (FliE)	WRNKWGYSSGVY	3	∆*aroA* ∆*asd* strains with mimotope-12 in pYA3342 vector
Mimotope-13 (LptD)	WTGPAYNTHSYR	6	∆*aroA* ∆*asd* strains with mimotope-13 in pYA3342 vector
Mimotope-14 (Hypothetical protein)	YSNPGLNDNFTG	2	∆*aroA* ∆*asd* strains with mimotope-14 in pYA3342 vector

**Table 3:** The treatments received by the chickens in the different experimental groups.

Group	Treatment	Route	Age at Vaccination (in Weeks)
1	∆*aroA* ∆*asd* strains with mimotope-9 and mimotope-10 in pYA3342 vector	Oral gavage	16 and 18
2	∆*aroA* ∆*asd* strains with mimotope-1 and mimotope-14 in pYA3342 vector	Oral gavage	16 and 18
3	∆*aroA* ∆*asd* strains with mimotope-11 and mimotope-12 in pYA3342 vector	Oral gavage	16 and 18
4	∆*aroA* ∆*asd* strains with mimotope-2 and mimotope-8 in pYA3342 vector	Oral gavage	16 and 18
5	∆*aroA* ∆*asd* strains with mimotope-6 and mimotope-7 in pYA3342 vector	Oral gavage	16 and 18
6	∆*aroA* ∆*asd* strains with mimotope-5 and mimotope-13 in pYA3342 vector	Oral gavage	16 and 18
7	∆*aroA* ∆*asd* strains with mimotope-3 and mimotope-4 in pYA3342 vector	Oral gavage	16 and 18
8	PBS (challenged positive group)	Oral gavage	16 and 18
9	PBS (unchallenged negative control group)	Oral gavage	16 and 18

**Table 4:** The comparative recovery of *C. hepaticus* from the bile and liver samples in vaccinated challenged groups and unvaccinated challenged positive control group.

Animal Group	Vaccine Strain Genotype Information	*C. hepaticus* Recovery Rate from Bile and Liver Samples Following Bacterial Culture (%)	Protection Rate (%)
1	APEC PSU ∆*aroA* ∆*asd* strains with mimotope-9 and mimotope-10 in pYA3342 vector	0%	100%
2	APEC PSU ∆*aroA* ∆*asd* strains with mimotope-1 and mimotope-4 in pYA3342 vector	0%	100%
3	APEC PSU ∆*aroA* ∆*asd* strains with mimotope-11 and mimotope-12 in pYA3342 vector	14.29%	85.71%
4	APEC PSU ∆*aroA* ∆*asd* strains with mimotope-2 and mimotope-8 in pYA3342 vector	0%	100%
5	APEC PSU ∆*aroA* ∆*asd* strains with mimotope-6 and mimotope-7 in pYA3342 vector	57.14%	42.86%
6	APEC PSU ∆*aroA* ∆*asd* strains with mimotope-5 and mimotope-13 in pYA3342 vector	0%	100%
7	APEC PSU ∆*aroA* ∆*asd* strains with mimotope-3 and mimotope-4 in pYA3342 vector	0%	100%
Control (unvaccinated, challenged)	PBS only	100%	0%

## Data Availability

The data supporting the findings of this study are available from the corresponding author upon request.

## Supplementary Materials

The following supporting information can be downloaded at: https://www.mdpi.com/article/10.3390/vaccines14040298/s1, Figure S1: Molecular construction and confirmation of recombinant *E. coli* DH5α Δ*ompC* strains expressing OmpC–mimotope fusion; Figure S2: Detection of *C. hepaticus* DNA from cultured bacterial isolates using a conventional PCR; Figure S3: Standard curves for absolute quantification of both host (beta-actin gene, *ACTB*) and *C. hepaticus* (glycerol kinase gene, *glk*); Table S1: List of fourteen identified *C. hepaticus* mimotopes as homologs of surface-localized proteins; Table S2: Summary of paired statistical analysis of IgG levels of pre-bleed and post vaccination; Table S3: Summary of statistical analysis of IgA levels of vaccinated groups-4, -6 and -7 and unchallenged unvaccinated control groups; Table S4: Descriptive statistics of *C. hepaticus* loads (mean log_10_ genome copies) in liver samples between vaccinated (Group-4 -6 and -7), challenged and unvaccinated and challenged (positive control) groups as measured by a quantitative real-time PCR; Table S5: Pairwise comparison of the mean log_10_ *C. hepaticus* genome copies in liver samples between vaccinated (Group-4 -6 and -7), challenged and unvaccinated and challenged (positive control) groups as measured by a quantitative real-time PCR; Table S6: Descriptive statistics of absolute *C. hepaticus* loads (mean log_10_ genome copies) in cecal contents between vaccinated (Group-4 -6 and -7), challenged and unvaccinated and challenged (positive control) groups as measured by a quantitative real-time PCR.

## Abbreviations

The following abbreviations are used in this manuscript:

APEC	Avian pathogenic *E. coli*
BGC	Bovine genital campylobacteriosis
ELISA	Enzyme-linked immunosorbent assay
GI	Gastrointestinal
IPTG	Isopropyl-β-D-1-thiogalactopyranoside
LB	Luria–Bertani
MCS	Multiple cloning site
MOMP	Major outer membrane protein
PNPP	p-nitrophenyl phosphate
qPCR	Quantitative polymerase chain reaction
NCBI	National center for biotechnology information
ORF	Open reading frame
OmpC	Outer membrane protein C
OMPs	Outer membrane proteins
PBS	Phosphate-buffered saline
SDS-PAGE	Sodium dodecyl sulfate–polyacrylamide gel electrophoresis
SLD	Spotty liver disease
SPF	Specific pathogen-free
TMH	Transmembrane helix
TNTC	Too numerous to count
X-gal	5-bromo-4-chloro-3-indolyl β-D-galactopyranoside
