# Supplementary material for: Identification and Expression of Immunogenic Mimotopes of C. hepaticus Using an E. coli-Based Surface Display System
Source: Vaccines (Basel). 2026 Mar 26;14(4):298. doi: 10.3390/vaccines14040298 (PMC13119680; doi:10.3390/vaccines14040298)
Supplement: Supplementary file 1 [file vaccines-14-00298-s001.zip › vaccines-4156416-supplementary.pdf]

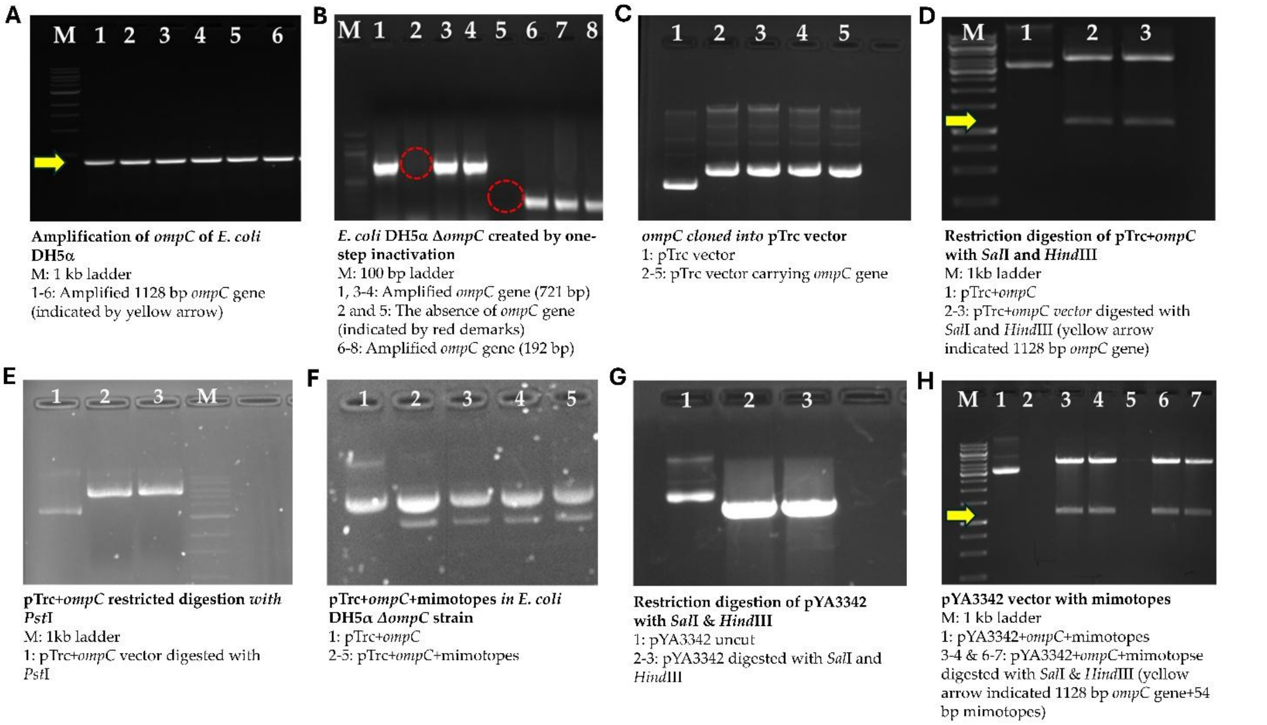

Figure S1. Molecular construction and confirmation of recombinant *E. coli* DH5*α* Δ*ompC* strains expressing OmpC–mimotope fusions. **(A)** PCR amplification of *ompC* (1128 bp) from *E. coli* DH5*α*. **(B)** Verification of *E. coli* DH5*α* Δ*ompC* mutant showing loss of *ompC* gene. **(C)** Cloning of *ompC* into pTrc vector. **(D)** Double digestion of pTrc+*ompC* with *Sal*I and *Hind*III confirms correct insertion. **(E)** Restriction digestion of pTrc + *ompC* with *Pst*I prior to mimotope cloning. **(F)** Confirmation of mimotope insertion into pTrc + *ompC* in Δ*ompC* strain. (G) Restriction digestion of pYA3342 vector with *Sal*I and *Hind*III. (H) Confirmation of pYA3342 + *ompC* + mimotope showing expected band size of 1182 bp (1128 bp of *ompC* + 54 bp mimotope).

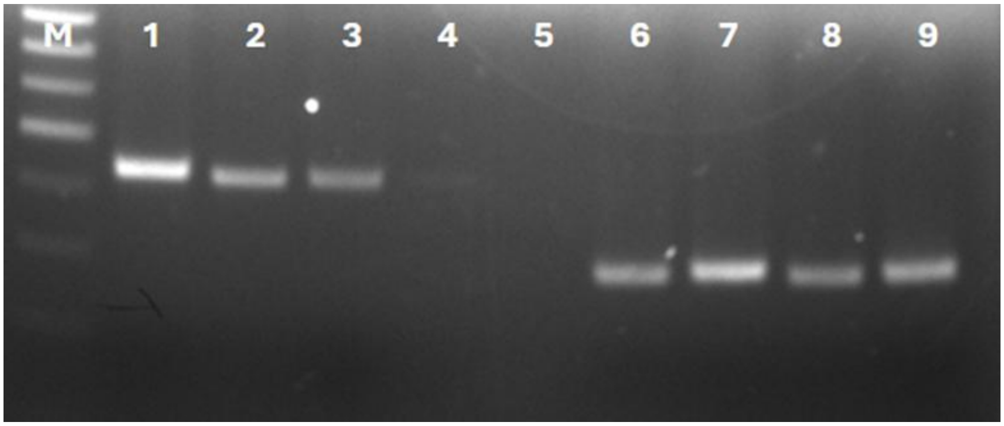

Figure S2. Detection of *C. hepaticus* DNA from cultured bacterial isolates using a conventional PCR. "M" denotes the 100 bp ladder (GeneRuler, Thermo Scientific). Lane 5 is a negative control. Both 463 bp (1-4

lanes) and 308 bp (6-9 lanes) correspond to two specific regions of the glycerol kinase (*glk*) gene of *C. hepaticus*.

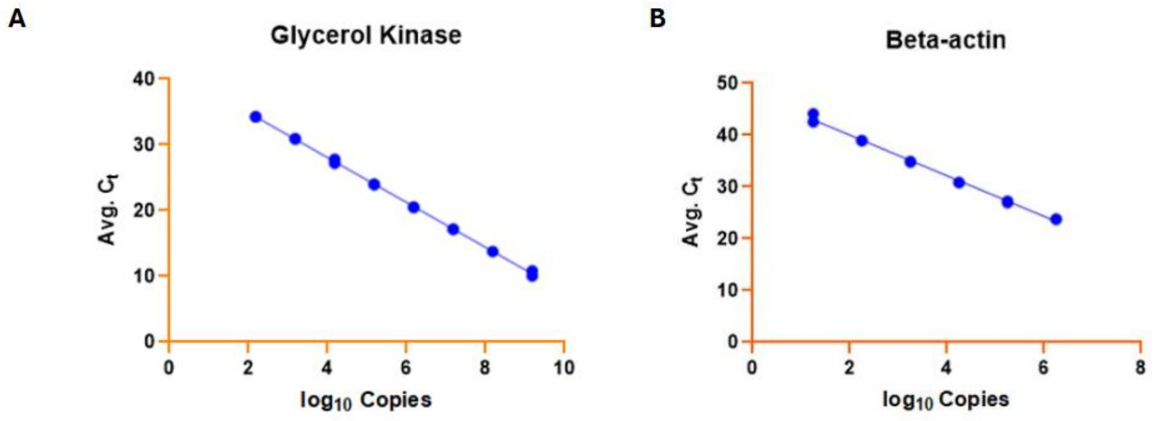

Figure S3. Standard curves for absolute quantification of both host (beta-actin gene, *ACTB*) and *C. hepaticus* (glycerol kinase gene, *glk*)

Table S1: List of fourteen identified *C. hepaticus* mimotopes as homologs of surface-localized proteins

Clone	Dodecapeptide	Name of the protein
1	EFYKHIKVTNKV	Hypothetical protein
2	GHWKHHFRPPAP	Flagellar hook-length control protein FliK
3	ISRWQKYPIYSS	Flagellar hook protein FlgE
4	MSELQVTVSNRA	Flagellin A
5	NHSNAWWSKW-R	Major outer membrane protein
6	NLSLTNADELAR	Hypothetical protein IRA69_03860
7	SSSHLHASNKHK	Hemagglutinin repeat-containing protein
8	SVRNQLGXXR-E	Flagellar basal body rod protein FlgB
9	SWGGVFRLANRY	Fibronectin-binding outer membrane protein CadF
10	TFAFADNFGHLR	Major outer membrane protein
11	WRAGPRHLVPSP	Flagellar hook-length control protein FliK
12	WRNKWGYSSGVY	LPS-assembly protein LptD
13	WTGPAYNTHSYR	LPS-assembly protein LptD
14	YSNPGLNDNFTG	Hypothetical protein

Table S2. Summary of paired statistical analysis of IgG levels of pre-vaccination and post-vaccination

Comparison	Group	Vaccine	Test used	Test statistics	Degree of freedom	p-value (one-tailed)
Pre-vac vs post-vac	4	Mimotope-2 (FliK)	Paired t-test	2.111	5	0.0212
Pre-vac vs post-vac	7	Mimotope-4 (Flagellin A)	Wilcoxon test	22	-	0.0391
Pre-vac vs post-vac	6	Mimotope-5 (MOMP)	Wilcoxon test	28	-	0.0078

Table S3. Summary of statistical analysis of IgA levels of vaccinated groups-4, -6 and -7 and unchallenged unvaccinated control groups

Comparison	Group	Vaccine	Test used	Test statistics	Number of pairs	p-value (one tailed)
Vaccinated vs control	4	Mimotope-2 (FliK)	Mann-Whitney	30	6	0.0179
Vaccinated vs control	7	Mimotope-4 (Flagellin A)	Mann-Whitney	29	7	0.0357
Vaccinated vs control	6	Mimotope-5 (MOMP)	Mann-Whitney	30	6	0.0179

Table S4. Descriptive statistics of *C. hepaticus* loads (mean $\log_{10}$ genome copies) in liver samples between vaccinated (Group-4 -6 and -7), challenged and unvaccinated and challenged (positive control) groups as measured by a quantitative real-time PCR

Group	Vaccine	Number of observations	Mean $\log_{10}$ load (±SD)
Positive control	-	5	1.253±0.452
Group 4	Mimotope-2 (FliK)	5	0.7565±0.261
Group 6	Mimotope-5 (MOMP)	6	1.035±0.298
Group 7	Mimotope-4 (Flagellin A)	5	0.2606±0.577

Table S5. Pairwise comparison of the mean $\log_{10}$ *C. hepaticus* genome copies in liver samples between vaccinated (Group-4 -6 and -7), challenged and unvaccinated and challenged (positive control) groups as measured by a quantitative real-time PCR

Comparison	Mean difference	Adjusted *p*-value
Group 4 vs. positive control	-0.4960	0.3036
Group 6 vs. positive control	-0.2172	0.8420
Group 7 vs. positive control	-0.9919	0.0110

Table S6. Descriptive statistics of absolute *C. hepaticus* loads (mean $\log_{10}$ genome copies) in cecal contents between vaccinated (Group-4 -6 and -7), challenged and unvaccinated and challenged (positive control) groups as measured by a quantitative real-time PCR

Group	Vaccine	Number of observations	Mean $\log_{10}$ load (±SD)
Positive control	-	5	0.4450±0.044
Group-4	Mimotope-2 (FliK)	5	0.4801±0.063
Group-6	Mimotope-5 (MOMP)	6	0.4334±0.03
Group-7	Mimotope-4 (Flagellin A)	5	0.4288±0.06
